# *Mannheimia haemolytica* and lipopolysaccharide induce airway epithelial inflammatory responses in an extensively developed ex vivo calf model

**DOI:** 10.1038/s41598-020-69982-0

**Published:** 2020-08-03

**Authors:** Yang Cai, Soheil Varasteh, Jos P. M. van Putten, Gert Folkerts, Saskia Braber

**Affiliations:** 1 Division of Pharmacology, Utrecht Institute for Pharmaceutical Sciences, Faculty of Science, Utrecht University, Utrecht, The Netherlands; 2 Department of Infectious Diseases and Immunology, Utrecht University, Utrecht, The Netherlands

**Keywords:** Infection, Acute inflammation, Bacterial infection, Respiratory tract diseases

## Abstract

Pulmonary infection is associated with inflammation and damage to the bronchial epithelium characterized by an increase in the release of inflammatory factors and a decrease in airway barrier function. Our objective is to optimize a method for the isolation and culture of primary bronchial epithelial cells (PBECs) and to provide an ex vivo model to study mechanisms of epithelial airway inflammation. PBECs were isolated and cultured from the airways of calves in a submerged cell culture and liquid–liquid interface system. A higher yield and cell viability were obtained after stripping the epithelium from the bronchial section compared to cutting the bronchial section in smaller pieces prior to digestion. *Mannheimia haemolytica* and lipopolysaccharide (LPS) as stimulants increased inflammatory responses (IL-8, IL-6 and TNF-α release), possibly, by the activation of "TLR-mediated MAPKs and NF-κB" signaling. Furthermore, *M. haemolytica* and LPS disrupted the bronchial epithelial layer as observed by a decreased transepithelial electrical resistance and zonula occludens-1 and E-cadherin expression. An optimized isolation and culture method for calf PBECs was developed, which cooperated with animal use Replacement, Reduction and Refinement (3R's) principle, and can also contribute to the increased knowledge and development of effective therapies for other animal and humans (childhood) respiratory diseases.

## Introduction

Respiratory diseases, such as pneumonia and bronchiolitis, are complex, multifactorial disorders caused by viral and/or microbial pathogens, an impaired immune system, as well as environmental and genetic factors. It is generally accepted that inflammation and damage of bronchial epithelium contribute to the development of respiratory diseases in animals and also humans^1–6^. The airway epithelium plays a central role in the maintenance of airway integrity and acts as a physical barrier to protect the lungs against inhaled (infectious) particles. Bacterial and viral infections can induce the release of inflammatory cytokines and chemokines, including IL-8, IL-6 and TNF-α from airway epithelial cells. The major cause of epithelial barrier breakdown during lung inflammation is related to the damage of tight and adherens junctions^7–10^.

Due to the risk of development and the presence of antibiotic resistance, new avenues have to be explored to tackle (opportunistic) infections. The primary culture of bronchial epithelial cells is of particular importance, allowing the characterization of pathogenic infection in airway epithelium, advancing our knowledge of airway inflammation, and developing new intervention strategies.

Because of the ethical and practical concerns, especially among vulnerable population groups, including children, it is highly challenging to obtain primary tissue for isolating primary bronchial epithelial cells (PBECs) from infants and children, which hinders progress in research. However, the bovine epithelium can be obtained on a regular basis in sufficient quantities from calves slaughtered for food consumption. Studies in calves have been shown predictive for respiratory infections for decades^11,12^. The calf model of respiratory syncytial virus (RSV) infection has been described as a relevant model for preclinical testing of vaccine candidates related to the similarities between human (h)RSV and bovine (b)RSV^12^. Related to the animal use Replacement, Reduction and Refinement (3R's) principle, primary airway epithelial cells from calves cultured ex vivo may be used as a respiratory disease model for investigating pathophysiological and immunological characteristics.

*Mannheimia haemolytica* is a Gram-negative bacterium associated with pneumonia in neonatal calves and is responsible for economic losses in the global livestock industry^13^. *M. haemolytica* produces several virulence factors, such as lipopolysaccharide (LPS) and flagellin, which play an important role in the pathogenesis of bovine pneumonia^14^. Acute pneumonia caused by *M. haemolytica* is characterized by a decline in the innate immune function, dysfunction of airway epithelium and a large influx of inflammatory factors into the airways^15,16^. Toll-like receptors (TLRs) play a major role in bacterial recognition and epithelial innate immunity, where TLR4 primarily recognizes endotoxin (LPS) and TLR5 recognizes bacterial flagellin^17,18^. Activation of TLRs by bacteria leads to TLR-mediated signal transduction pathways in epithelial cells (e.g., via phosphorylated MAPKs and NF-κB), and subsequent production of cytokines and chemokines that recruit and activate the innate and adaptive immune system and regulate the barrier function of epithelial cells. However, it is not well-described whether *M. haemolytica* can activate TLR4 and TLR5, impede normal epithelial barrier function and promote inflammation in an in vitro model with primary airway epithelial cells.

The aim of this study is to optimize a method for the isolation and culture of PBECs and to provide an ex vivo model to study mechanisms of epithelial airway inflammation induced by *M. haemolytica* and LPS. A detailed description of two isolation methods (stripping and cutting the bronchial section prior to digestion) of bovine PBECs was given. Thereafter, we examined the effect of *M. haemolytica* and LPS on cellular viability, the production of inflammatory factors, barrier function and the associated mechanisms in the PBEC model. *M. haemolytica* and LPS can induce the production of inflammatory factors (IL-8, IL-6 and TNF-α), and "TLR-mediated MAPKs and NF-κB" signal transduction may be one of the possible mechanisms of action. *M. haemolytica* and LPS reduced the transepithelial electrical resistance (TEER) and decreased expression of the tight junction protein, ZO-1 and adherens junction protein, E-cadherin.

## Results

### Establishment of primary cultures of calf bronchial epithelium

**Figure 1:**
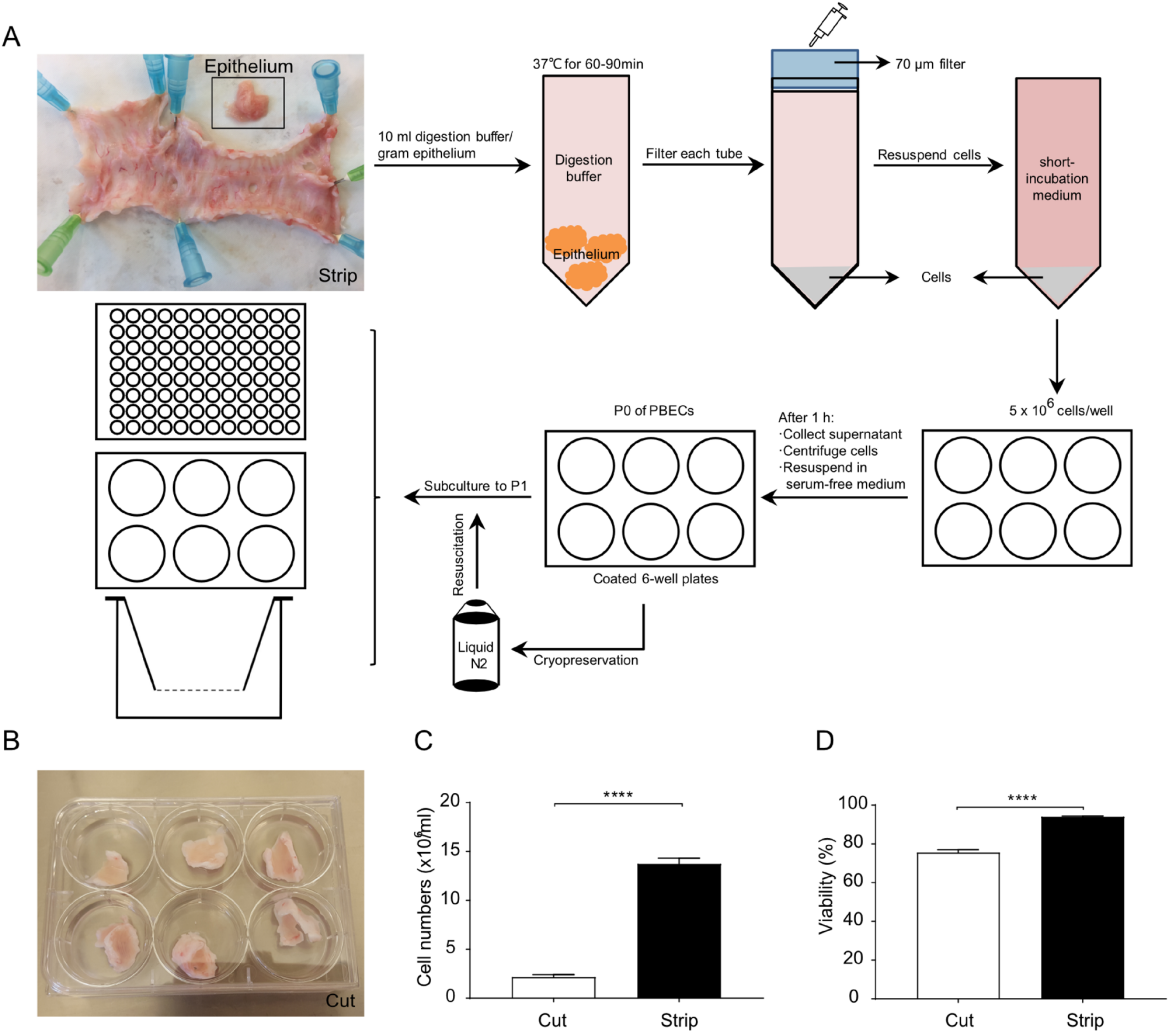
Establishment of primary cultures of calf bronchial epithelium. (**A**) Overview of isolation and culture of PBECs. The epithelium was first stripped from the bronchial section (**A**) or the bronchial section was cut into smaller fragments (**B**). After digestion, the total cell numbers (**C**) and cell viability (**D**) were significantly higher in the stripped bronchial epithelium compared to the bronchus that was cut into small fragments. ****P< 0.0001 (unpaired Student’s t-test). Data are presented as means ± SE (n = 15).

To better understand the respiratory infections in the calf, we first established an ex vivo calf bronchial epithelium infection model. Hereto, bronchial sections of a similar size and weight were cut from the primary bronchus of freshly slaughtered calves and subjected to the cell isolation procedure depicted in Fig. 1. One approach to isolate fresh PBECs involved stripping of the epithelium from the bronchial section followed by treatment with a digestion buffer (Fig. 1A, the strip method). Alternatively, the bronchial section was first to cut into smaller fragments and then subjected to enzymatic digestion (Fig. 1B, the cut method). Isolation of PBECs following the stripping of the epithelium resulted in a significantly higher yield and cell viability compared to the enzymatic digestion of complete bronchial explants (Fig. 1C, 2.2 ± 0.2 × 10^6^ cells/ml vs 13.7 ± 0.6 × 10^6^ cells/ml; D, 75.5 ± 1.6% vs 94.1 ± 0.3%; n = 15). Due to the high-efficiency characteristics, the strip method for isolating PBECs was used in all subsequent experiments.

**Figure 2:**
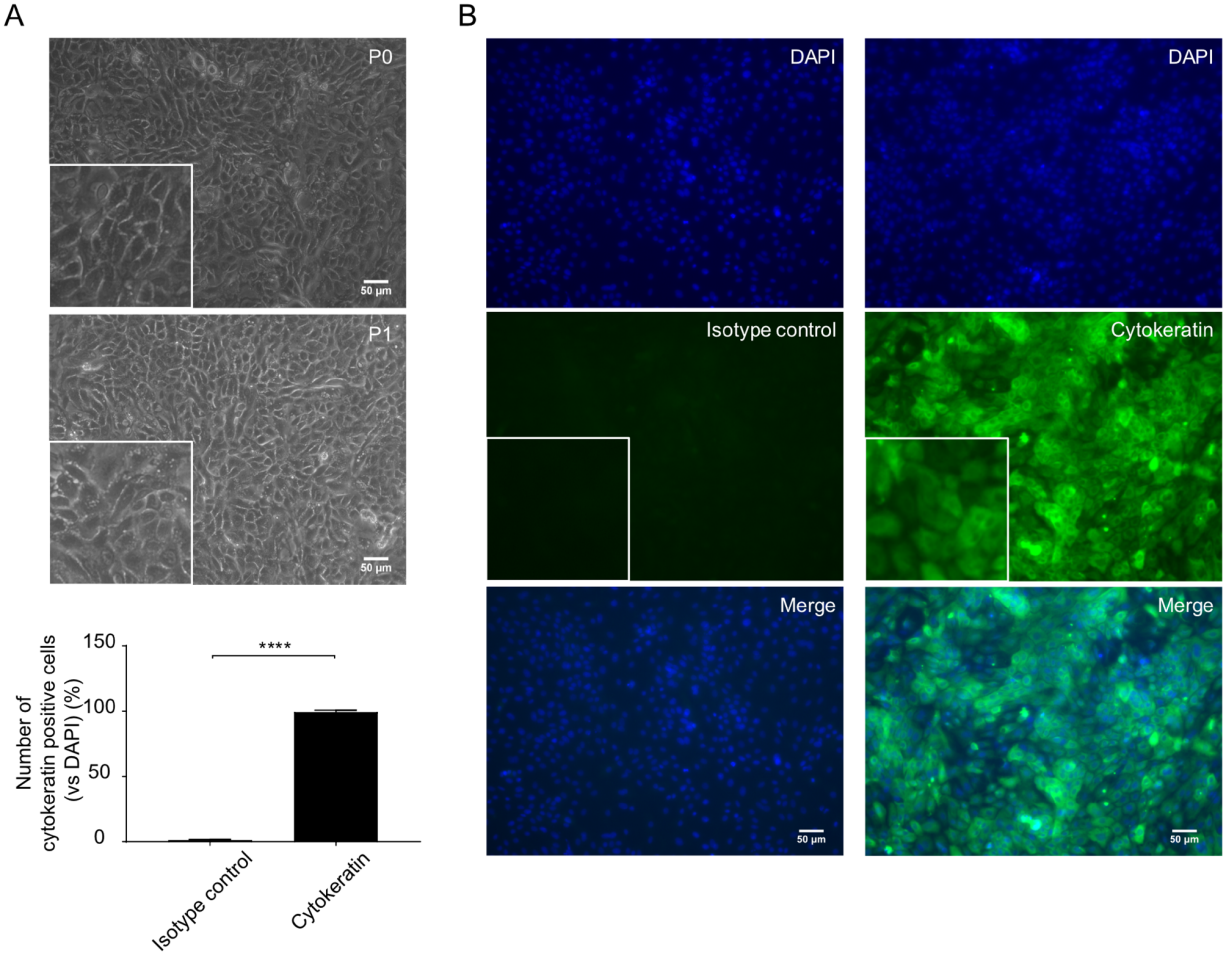
PBECs form a clear network with attached cytokeratin-positive cells. (**A**) Representative microscopic picture of *passage* 0 (P0) and *passage* 1 (P1) of PBECs in the SCC system. (**B**) P1 of PBECs stained by isotype control or cytokeratin antibody (green), followed by counterstain with 4′, 6-diamidino-2-phenylindole (DAPI), which illuminates cell nuclear material (blue). Original magnification, 200×; higher magnification, 400×. ****P<0.0001 (unpaired Student’s t-test). Data are presented as means ± SE (n = 5).

Isolated cells (obtained via the strip method) were incubated with short-incubation medium (1 h) and thereafter successfully propagated in serum-free medium (2–3 days). After 2–3 days, PBECs were cultured in FBS medium for another 2–3 days and formed clear networks of attached cells in the submerged cell culture (SCC) system. Both the initial and passaged PBECs exhibited a polygonal, cobblestone appearance, typical of epithelial cells as depicted in Fig. 2A. The epithelial lineage identity of the PBECs was confirmed by immunofluorescence staining of the cells for the epithelial marker cytokeratin. On average, 99.3 ± 1.4% (n = 6) of the PBECs stained positive for the expression of this cytokeratin (Fig. 2B).

### Exposure of PBECs towards *M. haemolytica* and LPS

**Figure 3:**
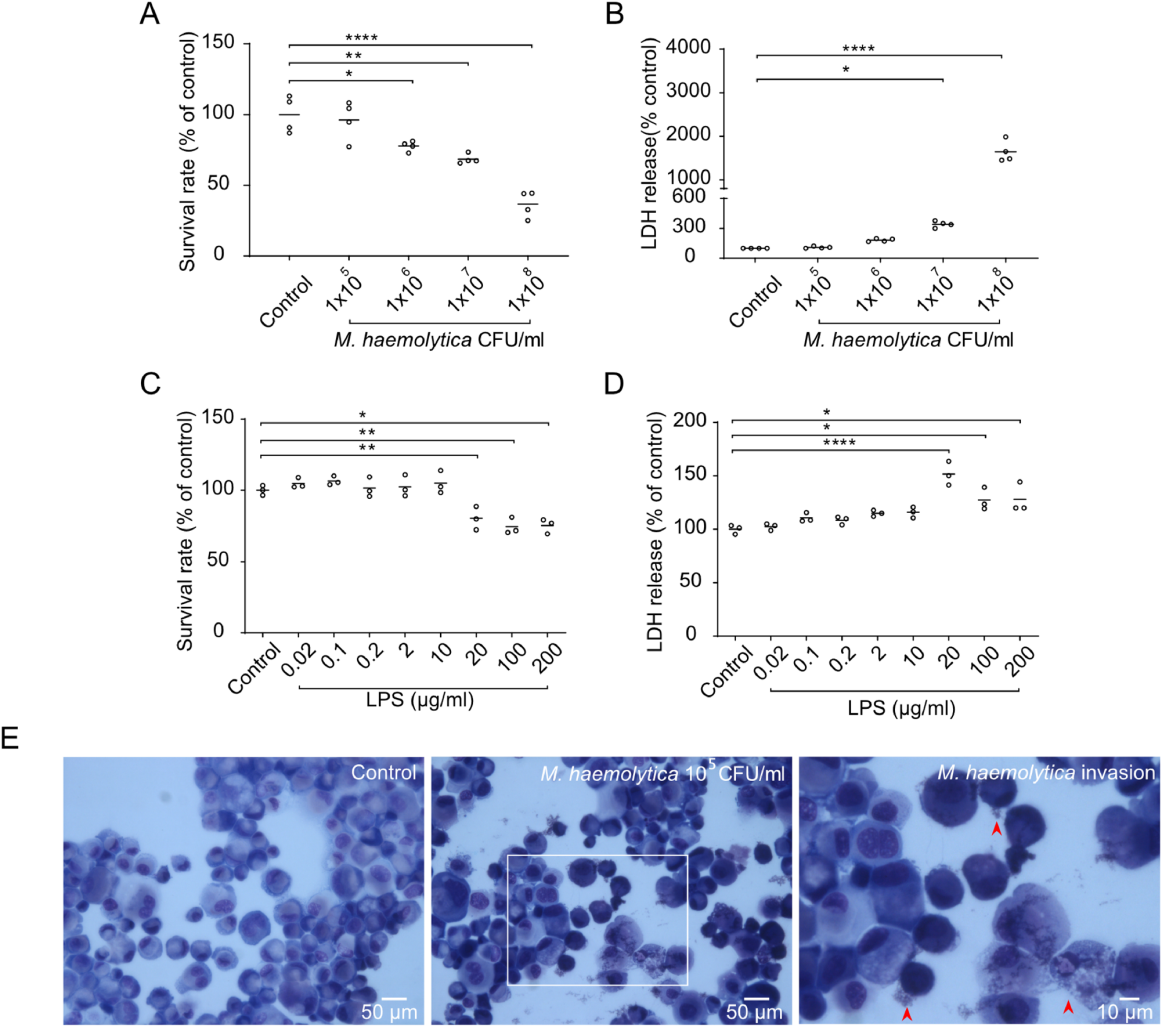
Exposure of PBECs towards *M. haemolytica* and LPS. *Passage* 1 (P1) of PBECs were incubated with increasing concentrations of *M. haemolytica* (1 × 10^5^–10^8^ CFU/ml) (**A–B**) and LPS (0.02–200 µg/ml) (**C-D**) for 24 h in the SCC system and (**A** and **C**) survival rates were determined by the percentage of MTT levels in control and LPS/*M. haemolytica*-treated PBECs. (**B** and **D**) LDH levels were measured in the supernatants of control and LPS/*M. haemolytica*-treated PBECs. (**E**) Representative microscopic pictures of P1 of control and *M. haemolytica*-treated PBECs on air-dried cytospin preparations. *P<0.05; **P<0.01; ****P<0.0001 (one-way ANOVA). Data are presented as means ± SE (n = 3 or 4).

To investigate the potential of the cultured PBECs as an infection model, we infected the cells with increasing concentrations of the respiratory pathogen *M. haemolytica.* Air-dried cytospin preparations with *M. haemolytica*-infected PBECs showed adhesion and invasion of *M. haemolytica* to the epithelial cells (Fig. 3E). To assess possible cell toxicity caused by *M. haemolytica*, cellular survival (MTT assay) and LDH release from the PBECs was measured after 24 h of infection. At *M. haemolytica* concentrations of > 10^5^ CFU/ml, cellular survival dropped in a concentration-dependent fashion in PBECs obtained from four different calves (Fig. 3A). This coincided with an increase in the LDH release (Fig. 3B). At concentration of LPS of > 10 µg/ml, the PBECs showed a lower survival rate (Fig. 3C) and a higher LDH release (Fig. 3D) for PBECs obtained from three different calves, resembling the toxic effect of the bacterial pathogen on the cells. Therefore, *M. haemolytica* concentrations of 10^5^ CFU/ml and 10 µg/ml LPS will be used in future experiments.

### Inflammatory pathways of PBECs activated by *M. haemolytica* and LPS

**Figure 4:**
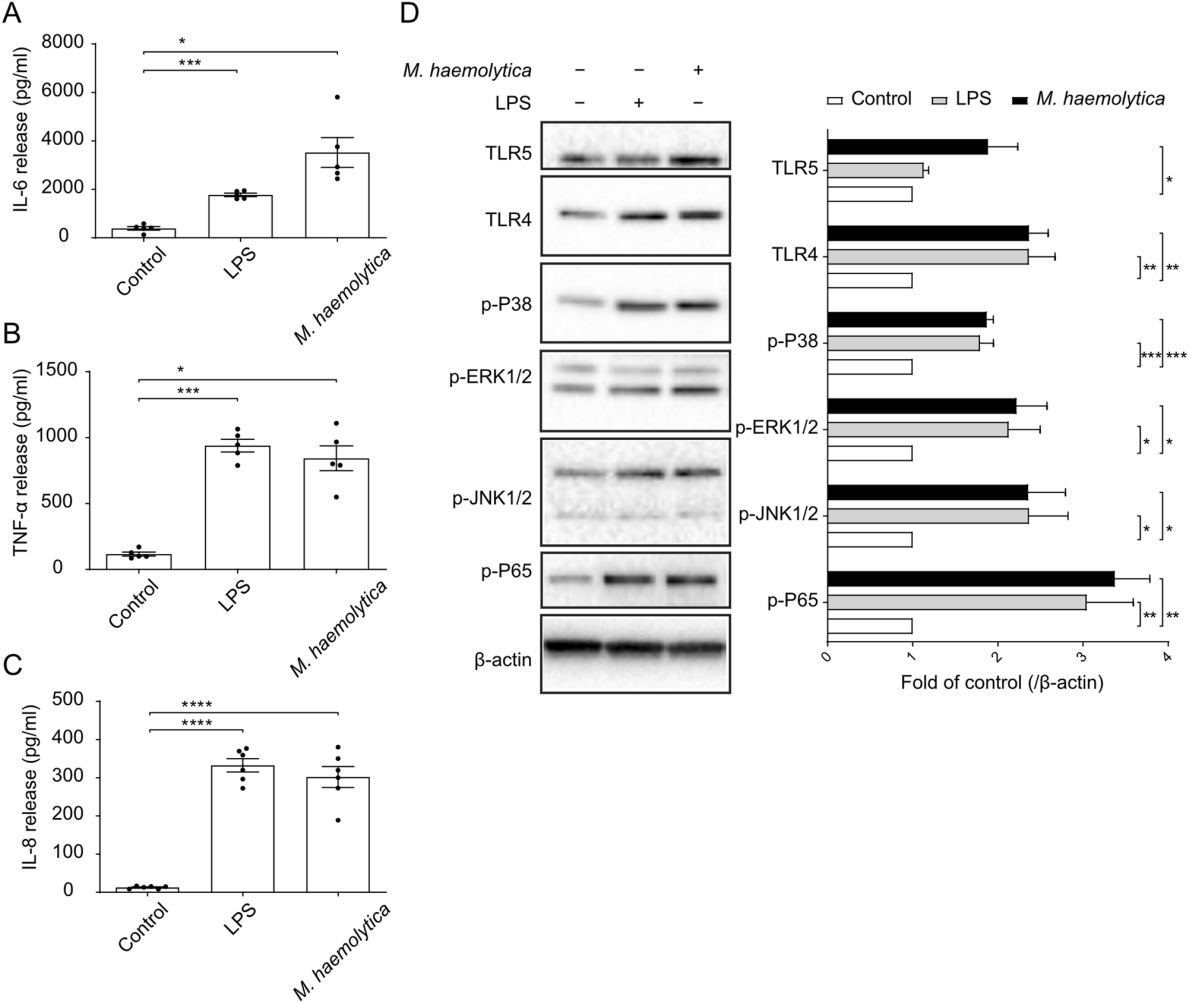
Inflammatory pathways of PBECs activated by *M. haemolytica* and LPS. *Passage* 1 (P1) of PBECs were incubated with LPS (10 µg/ml) and *M. haemolytica* (1 × 10^5^ CFU/ml) for 24 h in the SCC system and (**A**) interleukin (IL)-6, (**B**) tumor necrosis factor (TNF)-α and (**C**) IL-8 levels in the supernatants of control and LPS/*M. haemolytica*-treated PBECs were assessed by ELISA. (**D**) Expression of TLR4/5 and phosphorylation of p38, ERK1/2, JNK1/2 and NF-κB p65 were determined by western blot in control and LPS/*M. haemolytica*-treated PBECs (original blots are shown in Supplementary Figure S5A. *P<0.05; **P<0.01; ***P <0.001; (**A**–**C**) one-way ANOVA; and (**D**) two-way ANOVA. Data are presented as means ± SE (n = 5–6).

Infection of PBECs with *M. haemolytica* (1 × 10^5^, 1 × 10^6^, 1 × 10^7^ CFU/ml) increased the IL-8 production by the cells in a concentration-dependent fashion (Supplementary Figure S1). One of the factors that may contribute to IL-8 production is bacterial LPS. Addition of increasing concentrations (2–200 µg/ml) of purified LPS to the cultured PBECs indeed induced a concentration-dependent release of IL-8 in the cell culture supernatant (Supplementary Figure S2). The optimal concentrations of *M. haemolytica* (1 × 10^5^ CFU/ml) and LPS (10 µg/ml) as mentioned above did not only increase the IL-8 release (Fig. 4C) but also enhanced the IL-6 and TNF-α production by PBECs (Fig. 4A,B).

To learn more about the cellular pathways that are activated by *M. haemolytica* and LPS, we determined the expression of TLRs in the PBECs and the phosphorylation of MAPKs and p65 during exposure of the cells to the pathogen and LPS. Western blotting of cell lysates showed that the cultured PBECs express both TLR4 and TLR5 innate immune receptors. Infection of the cells with *M. haemolytica* increased both TLR protein levels, whereas LPS stimulation only resulted in a significant increase in TLR4 protein levels (Fig. 4D). On the other hand, both *M. haemolytica* and LPS promoted the phosphorylation of p38, ERK1/2, JNK1/2 MAPK and NF-κB p65 (Fig. 4D). Thus, the observed cellular release of the different pro-inflammatory mediators may result from a TLR-dependent activation of MAPKs and NF-κB p65 pathways in PBECs.

Moreover, stimulation of PBECs with flagellin (1, 10, 100, 1,000 ng/ml), a TLR5 agonist, increased the IL-8 production but did not affect the LDH release and survival rate. In addition, flagellin (10 ng/ml) also increased the TNF-α and IL-6 production by PBECs (Supplementary Figure S3).

### Effects of *M. haemolytica* and LPS on the barrier function of PBECs

**Figure 5:**
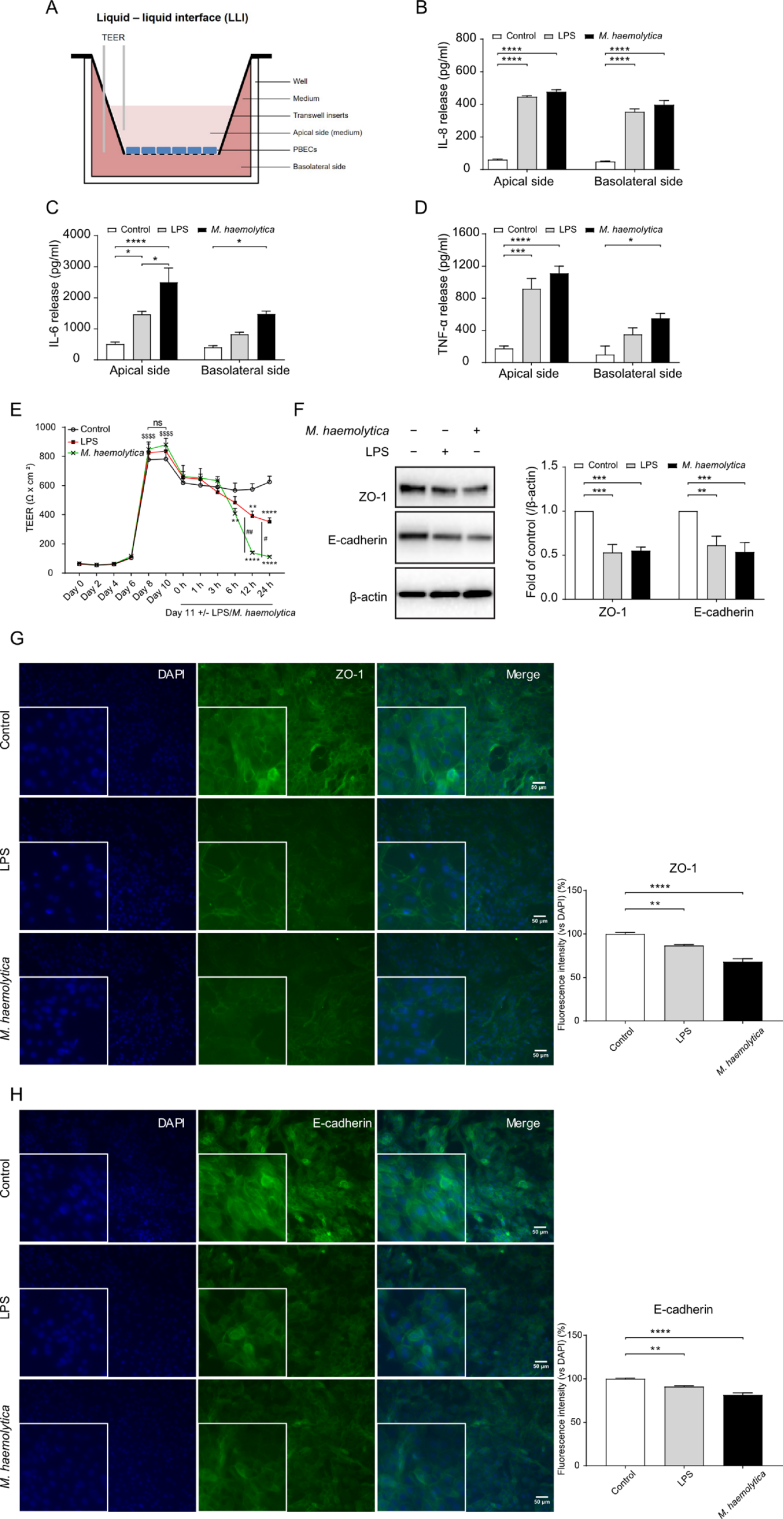
Effects of *M. haemolytica* and LPS on the barrier and immune function of PBECs. (**A**) P1 of PBECs were cultured on transwell inserts and FBS medium was added to both apical and basolateral compartments. (**B–D**) IL-8, IL-6 and TNF-α release in the supernatants of the apical and basolateral compartments from control and LPS/*M. haemolytica*-stimulated PBECs was measured by ELISA. (**E**) TEER value was measured every 2 days until day 10. At day 11, LPS (10 µg/ml) or *M. haemolytica* (1 × 10^5^ CFU/ml) were added to the apical side for 24 h and TEER value was measured at different time points (1 h, 3 h, 6 h, 12 h, and 24 h) in control and LPS/*M. haemolytica*-treated PBECs. (**F**) Expression of ZO-1 and E-cadherin were determined by western blot in control and LPS/*M. haemolytica*-treated PBECs (original blots are depicted in Supplementary Figure S5B). (**G** and **H**) P1 of PBECs were stained for ZO-1 (**G**) and E-cadherin (**H**) (green), followed by counterstaining with DAPI (blue). The fluorescence intensity was analyzed in representative pictures from PBECS obtained from six calves. Original magnification, 200×; higher magnification, 400×. $$$$*P*<0.0001 (Day 8 or 10 vs Day 6); #*P*<0.05; ##*P*<0.01 (*M. haemolytica* vs LPS group); **P<0.01; ***P<0.001; ****P<0.0001 (6, 12 or 24 h vs 0 h); (**B**–**F**) two-way ANOVA; and (**G-H**) one-way ANOVA. Data are presented as means ± SE (n = 4–6).

As in vivo*,* PBECs are part of a polarized monolayer of cells that form the bronchial lining, that may become disrupted during infection, we established PBEC growth on 0.4 µm semipermeable membranes in the liquid–liquid interface (LLI) system (Fig. 5A). In this system, it is possible to determine the integrity of the epithelial barrier and to differentiate between the apical and basolateral release of cyto/chemokines. As an indicator of epithelial barrier integrity, TEER was measured every two days of culture. After seeding the cells, TEER value started to increase from day 6 and values stabilized at around 600 Ω·cm^2^ on day 11 (Fig. 5E). The administration (day 11) of *M. haemolytica* (1 × 10^5^ CFU/ml) or LPS (10 µg/ml) to the apical compartment significantly decreased the TEER value after 6 h, 12 h, 24 h and 12 h, 24 h compared to the control PBECs, respectively (Fig. 5E). Especially, 12 h and 24 h M*. haemolytica* exposure induced a dramatic decrease in TEER value compared to the control and LPS-treated PBECs (Fig. 5E) even reaching the low TEER value after seeding. To learn more about this effect, the tight junction protein ZO-1 and the adherens junction protein E-cadherin were analyzed by Western blot and immunofluorescence staining. This showed that both *M. haemolytica* and LPS induced a significant decrease in the expression of the tight junction ZO-1 (Fig. 5F,G) and adherens junction E-cadherin (Fig. 5F,H).

### Differential release of IL-8, IL-6 and TNF-α by PBECs

To investigate whether exposure of polarized PBECs to the pathogen or LPS resulted in a differential release of cytokines into the apical or basolateral cell compartment, *M. haemolytica* (1 × 10^5^ CFU/ml) or LPS (10 µg/ml) were administered to the LLI system. After 24 h, the IL-8 release by PBECs at both the apical and basolateral compartments was significantly increased after exposure to both *M. haemolytica* and LPS (Fig. 5B). The IL-6 and TNF-α release by PBECs into the apical compartment was also significantly increased after both *M. haemolytica* and LPS stimulation. However, the release of IL-6 and TNF-α into the basolateral compartment was only significantly increased after stimulation with *M. haemolytica*, but not LPS (Fig. 5C,D).

After 24 h stimulation, LPS (10 µg/ml) did not affect the cellular survival (Supplementary Figure S4C) and LDH release in both apical (Supplementary Figure S4A) and basolateral (Supplementary Figure S4B) compartment of the PBECs. However, *M. haemolytica* (1 × 10^5^ CFU/ml) tended to decrease the cellular survival (Supplementary Figure S4C) and increase the LDH release in the apical compartment (Supplementary Figure S4A) but not in basolateral compartment (Supplementary Figure S4B), however these effects were not significantly different from control values.

## Discussion

**Figure 6:**
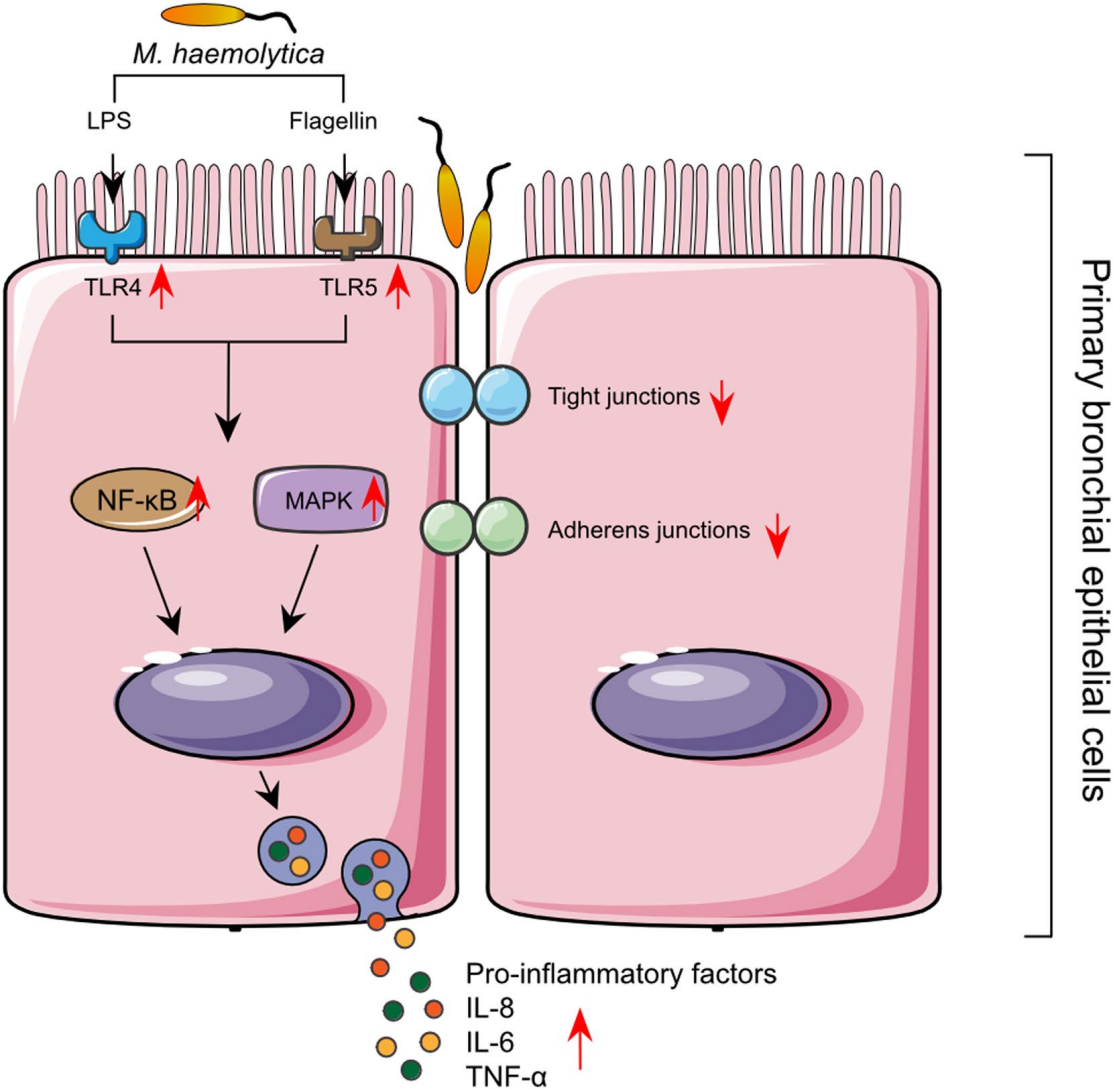
Schematic overview of the postulated mechanism of *M. haemolytica*-induced dysfunction of epithelial barrier and release of pro-inflammatory factors in PBECs. The two virulence factors of *M. haemolytica*, LPS and flagellin, induce the phosphorylation of MAPK proteins and NF-κB p65 by activating TLR4 and TLR5, respectively, and promote the release of pro-inflammatory factors (IL-8, IL-6 and TNF-α) in PBECs. The invasion of PBECs by *M. haemolytica* and the *M. haemolytica*-induced release of pro-inflammatory factors reduce the barrier function of epithelial cells, thereby weakening the resistance of epithelium to pathogens, and enhancing the development of inflammation in PBECs.

In the present study, different calf primary bronchial epithelial cell models were developed and optimized, which can be used to investigate the pathogenesis and mechanisms of respiratory diseases. First, the methodology of isolation and culturing PBECs was established and thereafter *M. haemolytica* and LPS and were used as stimulants to induce an in vitro inflammation/infection. Inflammation and damage of bronchial epithelium contribute to the development of respiratory diseases. Bacterial and viral pathogens causing these diseases can modulate the expression of TLRs and the release of inflammatory cytokines and chemokines from airway epithelial cells^5,7-9,19^. In this study, the inflammatory response of PBECs was tested via *M. haemolytica* and LPS exposure in two different cell culture systems. The effects of *M. haemolytica* and LPS on cellular viability, cytokine and chemokine release, barrier function and the associated mechanisms (TLRs, MAPKs, NF-κB) of the PBECs were examined (Fig. 6).

In the current study, fibroblast contamination was one of the largest problems during the isolation of calf PBECs, since, on one hand, fibroblasts grow faster than epithelial cells and leave limited area for the growth of epithelial cells, and on the other hand elimination of fibroblast contaminations from epithelial cultures are quite challenging. In the current study, stripping and cutting airway epithelium, were two possibilities to minimize the amount of connective tissue in the epithelial cell culture. Stripping epithelium was the most optimal way to improve the quantity and viability of PBECs (Fig. 1), probably due to the increase in the efficiency of digestion. Different studies cut the bronchus into smaller fragments and digest these fragments overnight^20^, whereas the method described in the current study, stripping epithelium, showed that more than 10 million PBECs can already be harvested after 1 h digestion. Fibroblasts can attach very quickly to culture plates, whereas PBECs need 2–3 days to attach^21,22^. To eliminate these fast-attaching fibroblasts, the cell population was first incubated in a short-incubation medium supplemented with 10% FBS for 1 h. Thereafter, serum-free medium (non-FBS) containing essential supplements (Table 1) was required for culture and attachment of PBECs^22^, resulting in a typical and uniform population of epithelial cells (Fig. 2A,B).

**Table 1:** Primary bronchial epithelial cell culture medium composition.

Composition	Cat. number	Serum-free medium	FBS medium (%)	Short-incubation medium (%)
RPMI-1640	BE12-702F	100%	100	0
DMEM	42430	0%	0	100
Fetal bovine serum	F7524	0%	10	10
Penicillin–streptomycin	P0781	1%	1	1
L-glutamine	25030-024	1%	1	1
MEM NEAA	11140-035	1%	1	1
Sodium selenite	S5261	4 ng/ml	0	0
Holo-transferrin	T1283	2.5 μg/ml	0	0
Epidermal growth factor	E9644	25 ng/ml	0	0
Insulin	I6634	1 μg/ml	0	0

In the present study, two different epithelial cell culture systems, including the SCC system and LLI system were optimized for investigating the *M. haemolytica* and LPS-induced inflammation and epithelial dysfunction in PBECs. These cell culture systems are extensively used and described in combination with other primary epithelial cells and cell lines^19,23,24^.

TLRs are particularly important in recognizing bacterial components in the airways, where endotoxin (LPS) can be recognized by TLR4 and flagellin can be recognized by TLR5^25,26^. Both LPS and flagellin are the main virulence factors of most human-related respiratory bacteria. However, there is not enough experimental evidence that *M. haemolytica,* or the virulence factors from *M. haemolytica,* flagellin and LPS, can induce expression of (epithelial) TLR4 and TLR5. It has been reported that bovine TLRs demonstrate a high sequence similarity with human TLR genes^27,28^. TLR4 and TLR5 receptors are abundantly expressed in airway epithelial cells of different species, such as cattle, sheep, mice, and humans^26,28–31^. In the present study, TLR4 and TLR5 protein levels increased 2.5 times and 2 times after stimulation with *M. haemolytica*, respectively, and both the TLR4 agonist (LPS) and TLR5 agonist (flagellin) induced an inflammatory response in PBECs (Fig. 4 and Supplementary Figure S3). It has been reported that the TLR4 mRNA expression increased 6 times and p38, ERK and NF-κB were activated in human airway epithelial cell line stimulated with *Klebsiella pneumoniae*^29^. Another study showed that the TLR5 mRNA expression increased 4 times in human airway epithelial cell line stimulated with *P. aeruginosa* flagellin^26^. Moreover, TLR4^-^/^-^ mice displayed an attenuated airway inflammatory response induced by *H. influenzae*, which was associated with a significant reduction in the clearance of this pathogen from the respiratory tract^32^. TLR5^-^/^-^ mice showed inhibition of MAPK phosphorylation and a decrease in inflammatory factors leading to a reduction of *P. aeruginosa*-induced lung infection. The TLR5–TLR4 cooperation is important for effective immune responses to lung infection^33^.

"TLR-mediated MAPKs and NF-κB" signaling pathways could play a role in the *M. haemolytica* and LPS-induced inflammation. It is known that the MAPK pathway, including extracellular signal-regulated kinase 1/2 (ERK1/2), p38, and c-Jun NH2-terminal kinase (JNK), as well as the NF-κB pathway are involved in the regulation of the synthesis of inflammatory mediators^34^. In general, after activation of TLR4 and TLR5, the classical "TLR-mediated MAPKs and NF-κB" signaling cascade will be activated, and NF-κB and MAPK signaling immediately promote the release of cytokines (especially IL-6 and TNF-α) and chemokines (especially IL-8), to recruit immune cells (e.g., macrophages, neutrophils) into the airway to eliminate bacteria^25^. The chemokine IL-8 and cytokines IL-6 and TNF-α have a wide range of effects on different cell types and are involved in the inflammation of respiratory diseases^9,19,35-37^. In our study, PBECs stimulated with *M. haemolytica* or LPS showed an increased expression of TLR4 and TLR5, while MAPK (p38, ERK1/2, JNK1/2) and NF-κB p65 were activated, possibly leading to the production of inflammatory cytokines (L-6, TNF-α) and chemokines (IL-8) (Fig. 4,5 and 6). In agreement with our study using undifferentiated PBECs, Cozens et al. found that *M. haemolytica* invades differentiated bovine airway epithelial cells by transcytosis and induces IL-1β, IL-6, TNF-α and IL-8 release by these epithelial cells^37^. It has also been reported that super-infection of human bronchial epithelial cells by *Staphylococcus aureus* leads to an enhanced phosphorylation of the p38 MAPK as well as an increased activation of the MAPKs ERK1/2 and JNK^38^. In addition, in vivo and in vitro studies showed that *H. influenzae* and *S. pneumoniae* disrupt airway epithelial barrier integrity by stimulating TLR2/4 and by activating the p38 MAPK and TGF-β pathways^39^.Moreover, TLR5 activation in response to bacterial flagellin may result in impaired epithelial barrier integrity and increased intestinal inflammation^40^.

In the present study, the effect of *M. haemolytica* and LPS on the airway barrier function of PBECs was investigated and the cultured PBECs created apical junctional complexes formed by tight and adherens junctions resulting in a tight network after culturing in the LLI system for 8–11 days (Fig. 5E,G,H). The peak TEER value was 800 ± 100 Ω·cm^2^ measured at day 10 (Fig. 5E), which is comparable to the TEER values (700–1,200 Ω·cm^2^) of human tracheal and bronchial epithelial cells obtained from healthy donors^41,42^. Cozens et al. reported that a peak TEER value from 500 to 1,400 Ω cm^2^ was measured in a LLI system with airway epithelial cells derived from different donor calves after approximately 5 days of culture and these cells showed the high intensity of ZO-1 staining after culturing for 11 days in transwells, which is similar to our observations in the LLI system^43^. The air–liquid interface (ALI) culture of human bronchial epithelial cells indicated a maximal TEER value of 766 ± 154 Ω cm^2^ 7 days after seeding^44^. In the present study, TEER value dropped to approximately 600 Ω.cm^2^ after reaching maximal TEER value at Day 10 (800 ± 100 Ω·cm^2^), while at day 11 the TEER value stabilized (Fig. 5E). Another study also showed that the TEER of human nasal epithelial cells cultured in a LLI system dropped to approximately 500 Ω.cm^2^ after reaching the maximum TEER value (3,133 ± 665 Ω.cm^2)45^. The drop in TEER values before stabilization could be related to the beginning of cell senescence^46^ or differentiation state of the cells^43^.

When TEER value stabilized (day 11), *M. haemolytica* and LPS were administered to the apical side of the LLI system and a significant decrease of TEER and lower expression of ZO-1 and E-cadherin was observed after *M. haemolytica* and LPS incubation for 24 h, which indicated a breakdown of junctional complexes resulting in dysfunction of the bronchial epithelial barrier. In particular, the TEER value started to decrease after 6 h *M. haemolytica* stimulation, and after 12 h the TEER value mimicked the value observed after seeding the PBECs, which may imply a dramatic disruption of the airway epithelial barrier, which will promote the rapid invasion of *M. haemolytica* through the epithelium to other tissues and blood vessels, aggravating the severity of acute lung infections. Cozens et al. found that the TEER values of bovine airway epithelial cells started to decrease after *M. haemolytica* stimulation for 24 h, but a significant decrease occurred after 48 h stimulation^37^. Differences between our observations and the study of Cozens et al. could be related to the difference between, the LLI and ALI model, differentiation state of the airway epithelial cells, *M. haemolytica* isolates, and the number of CFU.

A significant increase in IL-8 release at both apical and basolateral compartments of the transwell inserts was detected after *M. haemolytica* and LPS exposure (Fig. 5B). The anti-IL-8 antibody was already be proved to reduce lung epithelial injury and protect the alveolar epithelial barrier after acid-induced lung injury^47,48^. Moreover, IL-6 and TNF-α release were significantly increased at the apical and basolateral compartments of the transwell insert after exposure to *M. haemolytica* (Fig. 5C,D), while LPS only increased the apical IL-6 and TNF-α release by PBECs. In our model, *M. haemolytica* tended to affect the cell viability of PBECs in the LLI system (Supplementary Figure S4), which may result in lower TEER values (Fig. 5E) and higher IL-6 and TNF-α release (Fig. 5C,D) after infection. *M. haemolytica* produces besides LPS and flagellin, also another important virulence factor, leukotoxin, which could also contribute to the observed destructive effects on the airway epithelial layer^15^.

Chemokines and cytokines produced by epithelial cells infected with *M. haemolytica* have the ability to recruit and activate cellular innate and adaptive immune cells and regulate airway epithelial barrier function, which can stimulate autophagy, phagocytosis, and clearance of necrotic cells and pathogens, further contributing to the inflammatory responses^9^. Furthermore, the release of IL-8, IL-6 and TNF-α at the basolateral compartment simulate the release of inflammatory factors from the epithelium into the bloodstream observed in animal and human (childhood) respiratory diseases.

In summary, an optimized culture method for calf PBECs was developed and LPS/*M. haemolytica*-induced inflammatory responses in two different systems (SCC and LLI systems) with PBECs were detected. In addition, a possible mechanism for the induction of cellular inflammation and a decrease of barrier function in epithelial cells induced by *M. haemolytica* or LPS was given. Our data showed that *M. haemolytica* and LPS significantly increases IL-8, IL-6 and TNF-α release and decrease bronchial epithelial barrier function in PBEC, possibly by activating "TLR-mediated MAPKs and NF-κB" signaling pathways (Fig. 6). Although this is an ex vivo model with PBECs from calves, the *M. haemolytica*/LPS-induced inflammation, TLR4/5 expression and lung barrier dysfunction are mimicking important features and mechanisms of respiratory diseases which are frequently observed in other animals and human (children) from which it is not easy to obtain and culture bronchial epithelial cells. The development of these ex vivo models with PBECs will reduce the use of in vivo studies and will contribute to the principles of Replacement, Reduction and Refinement (3R's)^49^. These PBEC culture systems could be used to investigate the pathogenesis and intervention strategies of respiratory diseases.

## Methods

### Isolation and culture of primary bronchial epithelial cells

PBECs were isolated from bovine lung epithelium obtained from the lungs of freshly slaughtered calves aged 6–8 months provided by Ekro bv (Apeldoorn, The Netherlands). Fresh lungs were always kept on ice during transport to the laboratory. Thereafter, the lungs were washed with PBS (Lonza, Verviers, Belgium) containing penicillin (100 U/ml, Sigma-Aldrich, St. Louis, MO) and streptomycin (100 μg/ml, Sigma-Aldrich) before and after removing the surrounding tissue from the right or left primary bronchus. The bronchus was opened with sterile scissors. [1] The bronchus was cut into smaller pieces or [2] the bronchus was pinned on foam board with sterile needles and bronchial epithelium was carefully stripped from the primary bronchus by using tweezers. For both methods, the similar size and weight of the bronchus was used. The small bronchial pieces [1] or the bronchial epithelium [2] was digested in Dulbecco’s modified Eagle’s medium (DMEM) containing pronase (1 mg/ml, Sigma-Aldrich), deoxyribonuclease I (500 μg/ml, Sigma-Aldrich), penicillin (100 U/ml, Sigma-Aldrich) and streptomycin (100 μg/ml, Sigma-Aldrich) for 60–90 min at 37 °C and was shaken 3 times during this procedure. Digestion was stopped by adding a triple volume short-incubation medium (Table 1). Epithelium debris was removed by passing the tissue through a 70 μm cell strainer (Corning, New York, USA). Cell suspensions were centrifuged at 280×*g* for 15 min at 4 °C and resuspended in short-incubation medium (1 × 10^6^ cells/ml). These cell suspensions were transferred to 6-well plates (5 ml/well, Corning) and incubated at 37 °C in a humidified atmosphere of 95% air and 5% CO_2_ for 1 h. Supernatants were gently collected and centrifuged at 280×*g* for 5 min, resuspended in serum-free medium (Table 1) and added to 6-well plates pre-coated with collagen (30 μg/ml, Advanced BioMatrix, San Diego, CA) in combination with fibronectin (10 μg/ml, Sigma-Aldrich), and bovine serum albumin (BSA; 10 μg/ml, Sigma-Aldrich).

### Submerged cell culture (SCC) system

PBECs (from *passage* 0) in serum-free medium were grown at 37 °C in a humidified atmosphere of 95% air and 5% CO_2_ for 2–3 days until reaching near-confluence (70–90%) and forming clear network structures. After these 2–3 days, serum-free medium was changed into 10% FBS medium and cells were grown for another 2–3 days.

To subculture the adherent PBECs, PBECs were washed twice with pre-warmed PBS (37 °C), detached and passaged using 0.05% trypsin–EDTA (Gibco, ThermoFisher Scientific, Waltham, MA). The *passage* 1 (P1) of PBECs was centrifuged at 280×*g* for 5 min and suspended in FBS medium (Table 1) in 6- or 96-well plates for the following experiments.

### Liquid–liquid interface (LLI) system

P1 of the PBECs suspension (1 × 10^6^ cells/ml, FBS medium, 300 µl) was added to the apical compartment of the permeable 0.3 cm^2^ high pore density polyethylene membrane transwell inserts (353,495, Corning) placed in a 24-well plate and 700 μl FBS medium was added to the basolateral compartment. The PBECs were incubated at 37 °C in a humidified atmosphere of 95% air and 5% CO_2_. TEER of PBECs was measured by a Millicell-ERS Volt-Ohm meter (Millipore, Merck, Darmstadt, Germany) every 2 days. The culture medium from the basolateral and apical compartment was refreshed after TEER measurement and experiments started at day 11 when TEER values of about 600 Ω·cm^2^ were achieved.

### Cell count and viability

Cell counts and differential cell analyses were performed on the PBEC suspension after removing debris by passing through a 70 μm cell strainer. Differential cell counts were determined by Diff-Quick (Medion Diagnostics International Inc., FL, USA) staining on cytospin preparations and a minimum of 500 cells were counted. The viability of PBECs was assessed by trypan blue dye exclusion (0.2%, Sigma-Aldrich, Zwijndrecht, the Netherlands).

### Bacterial growth conditions

*M. haemolytica* (isolated from a pneumonic bovine lung) was kindly provided by Jos van Putten (Utrecht University, The Netherlands). *M. haemolytica* was incubated overnight at 37 °C in sheep blood agar (Biotrading, Mijdrecht, The Netherlands).

### LPS, flagellin and bacterial ex vivo stimulation

P1 of PBECs were cultured at a density of 1 × 10^6^ cells/ml in 96- or 6-well plates pre-coated with collagen, fibronectin and BSA or in non-coated transwell inserts. After reaching near-confluence and stable TEER value, these PBECs were stimulated with commercially available LPS (isolated from *E.coli O111:B4*, Sigma-Aldrich) or flagellin (isolated from *P. aeruginosa*, Invivogen, CA, USA) or *M. haemolytica* for 24 h and supernatants were collected and stored at -20 °C until analysis.

### Lactate dehydrogenase (LDH) assay

P1 of PBECs were grown in 96-well plates or in transwell inserts as described above and the cytotoxic effect of LPS, *M. haemolytica* or flagellin on the PBECs was evaluated by measuring LDH leakage. LDH was measured in the supernatants using the CytoTox 96 nonradioactive cytotoxicity assay kit (Promega Corp., Madison, WI, USA) according to manufacturer's instructions.

### 3-(4,5-dimethyl-2-thiazolyl)-2,5-diphenyl-2H-tetrazolium bromide (MTT) assay

P1 of PBECs were grown in 96-well plates or in transwell inserts as described above and the viability of cells was measured using MTT assay. MTT (Sigma-Aldrich) was dissolved at a final concentration of 0.5 mg/mL in FBS medium. Each culture well was delicately washed with pre-warmed (37 °C) PBS before adding a 120 µl MTT solution. After 3 h incubation (37 °C, 5% CO_2_), the formed formazan crystals were dissolved in 100 μl of dimethyl sulfoxide (DMSO, Sigma-Aldrich) and absorbance was read at 595 nm using a microplate reader (Bio-Rad Laboratories, Hercules, CA).

### ELISA measurement

P1 of PBECs were grown in 96-well plates or in transwell inserts as described above. The inflammatory response of PBECs after LPS, *M. haemolytica,* or flagellin stimulation was determined by measuring IL-8 (Mabtech, Nacka Strand, Sweden), IL-6 (Invitrogen, ThermoFisher Scientific) and TNF-α (R&D Systems, Minneapolis, MN) in supernatants using ELISA according to manufacturer's instructions. The absorbance was measured at 450 nm using a microplate reader (Bio-Rad Laboratories).

### Western blotting

P1 of PBECs (1 × 10^6^ cells/ml) were incubated with LPS (10 µg/ml) or *M. haemolytica* (1 × 10^5^ CFU/ml) at 37 °C for 24 h in both SCC and LLI systems, thereafter the supernatant was aspirated, and cells were washed 3 times with PBS. After this, total cell lysates were prepared by adding RIPA cell lysis buffer (ThermoFisher Scientific) containing protease inhibitors (Roche Applied Science, Pennsburg, Germany). Total protein content was estimated by bicinchoninic acid (BCA) analysis (Pierce, ThermoFisher Scientific) according to the manufacturer’s protocol. 30 μg of protein sample was loaded onto polyacrylamide gels (4–20% Tris–HCl, Bio-Rad Laboratories), separated using electrophoresis, and electrotransferred onto polyvinylidene difluoride membranes (Bio-Rad Laboratories). The membranes were blocked with PBS containing 0.05% Tween-20 (PBST) and 5% milk proteins for 1 h at room temperature and incubated with primary antibodies at 4 °C overnight (TLR5, 1:250, #sc57461, Santa Cruz Biotechnology, Dallas, TX; TLR4, 1:1000, #pa5-23284, ThermoFisher Scientific; p-p38, 1:1000, #9215; p-ERK1/2, 1:1000, #9101; p-JNK1/2, 1:1000, #9251; p-p65, 1:1000, #3033; β-actin, 1:5000, #4970, Cell Signaling Technology, Beverly, MA; ZO-1, 1:1000, #339100, Invitrogen, ThermoFisher Scientific; E-cadherin, 1:2000, #610182, BD Biosciences), followed by washing blots in PBST. Appropriate horseradish peroxidase-coupled secondary antibodies from Dako (Agilent Technologies, Santa Clara, CA) were applied for 1 h. Membranes were incubated with ECL western blotting substrates (Bio-Rad Laboratories) prior to obtaining the digital images. Digital images were acquired with the Molecular Imager Gel Doc XR system (Bio-Rad Laboratories) and analyzed with Image Lab 5.0 (Bio-Rad Laboratories).

### Immunofluorescence

P1 of PBECs suspension (1 × 10^6^ cells/ml) were grown in 6-well plates (5 ml/well) for 2–3 days until reaching near-confluence (70–90%) or in transwell inserts for 8–11 days until forming stable TEER values as described above. Immunofluorescence staining was conducted to detect the epithelial marker cytokeratin, the tight junction protein ZO-1 and the adherens junction protein E-cadherin. PBECs were fixed with 10% formalin (Baker, Deventer, The Netherlands) and after washing with PBS, the cells were permeabilized with PBS containing 0.1% (v/v) Triton-X-100 for 5 min, followed by blocking with 5% serum in 1% (w/v) BSA/PBS for 30 min at room temperature. Thereafter, PBECs were incubated overnight with primary anti-wide spectrum cytokeratin antibody (1:50, #ab9377, Abcam, Cambridge, UK), ZO-1 (1:50, #339100, Invitrogen, ThermoFisher Scientific) and E-cadherin (1:50, #610182, BD Biosciences, USA) followed by incubation with Alexa-Fluor conjugated secondary antibodies (Invitrogen, ThermoFisher Scientific) for 1 h at room temperature in dark. Nuclear counterstaining was performed with DAPI containing anti-fade reagent (ready to use, Invitrogen, ThermoFisher Scientific). Cytokeratin, ZO-1 and E-cadherin were visualized, and images were taken using a fluorescence microscope (Keyence BZ-9000, Osaka, Japan). Fluorescence intensity was quantified by ImageJ (Version 1.8.0, National Institutes of Health, USA) and presented as fluorescence intensity (vs DAPI). In addition, cell numbers of cytokeratin were counted by ImageJ and expressed as a percentage of cytokeratin-positive cells (vs DAPI).

### Statistical analysis

Experimental results were expressed as means ± SE. Statistically significant differences between groups were determined by unpaired Student’s t-test, one-way or two-way ANOVA using GraphPad Prism (version 7.0). Results were considered statistically significant when P < 0.05.

## Supplementary information

Supplementary information.

## Data Availability

Data sharing is not applicable to this article as no datasets were generated or analyzed during the present study.
