# Supplementary material for: Mannheimia haemolytica and lipopolysaccharide induce airway epithelial inflammatory responses in an extensively developed ex vivo calf model
Source: Sci Rep. 2020 Aug 3;10:13042. doi: 10.1038/s41598-020-69982-0 (PMC7400546; doi:10.1038/s41598-020-69982-0)
Supplement: Supplementary file 1 — Supplementary information. [file 41598_2020_69982_MOESM1_ESM.pdf]

Mannheimia haemolytica and lipopolysaccharide induce airway epithelial inflammatory responses in an extensively developed *ex vivo* calf model

Yang Cai[1], Soheil Varasteh[1], Jos P. M. van Putten[2], Gert Folkerts[1], and Saskia Braber[1]

[1] Division of Pharmacology, Utrecht Institute for Pharmaceutical Sciences, Faculty of Science, Utrecht University, Utrecht, The Netherlands

[2] Department of Infectious Diseases and Immunology, Utrecht University, Utrecht, The Netherlands

Corresponding author:

Dr. Saskia Braber

Utrecht University, Department of Pharmaceutical Sciences, Division of Pharmacology

Universiteitsweg 99, 3584 CG, Utrecht, The Netherlands

Telephone: +31(0)622483913

Email : S.Braber@uu.nl

Supplementary figures

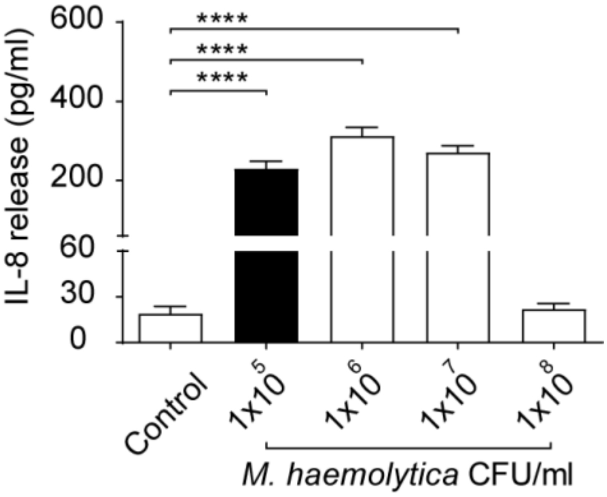

Figure S1. *M. haemolytica* increased IL-8 production by PBECs. *Passage* 1 (P1) of PBECs were incubated with increasing concentrations of *M. haemolytica* (1 x10^5-10^8 CFU/ml) for 24 h in the submerged cell culture (SCC) system and interleukin (IL)-8 levels in the supernatants of control and *M. haemolytica*-treated PBECs were assessed by ELISA. ****P<0.0001 (one-way ANOVA). Data are presented as means ± SE (n=4).

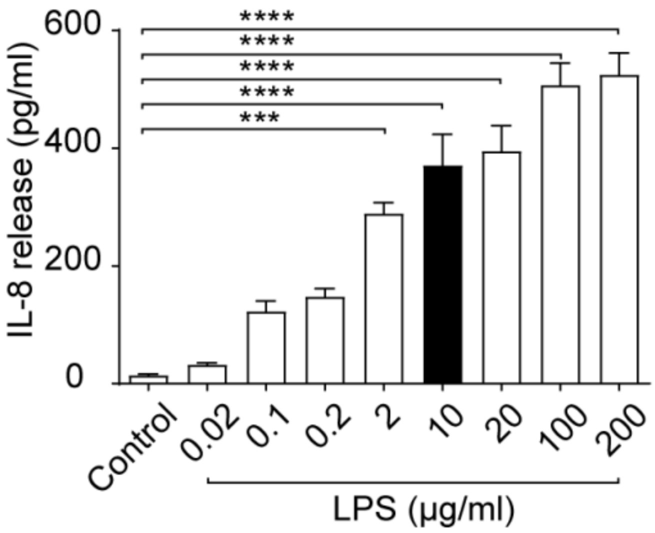

Figure S2. LPS increased IL-8 production by PBECs. *Passage* 1 (P1) of PBECs was incubated with increasing concentrations of LPS (0.02-200 µg/ml) for 24 h in the submerged cell culture (SCC) system and interleukin (IL)-8 levels in the supernatants of control and LPS-treated PBECs were assessed by ELISA. ***P<0.001, ****P<0.0001 (one-way ANOVA). Data are presented as means ± SE (n=3).

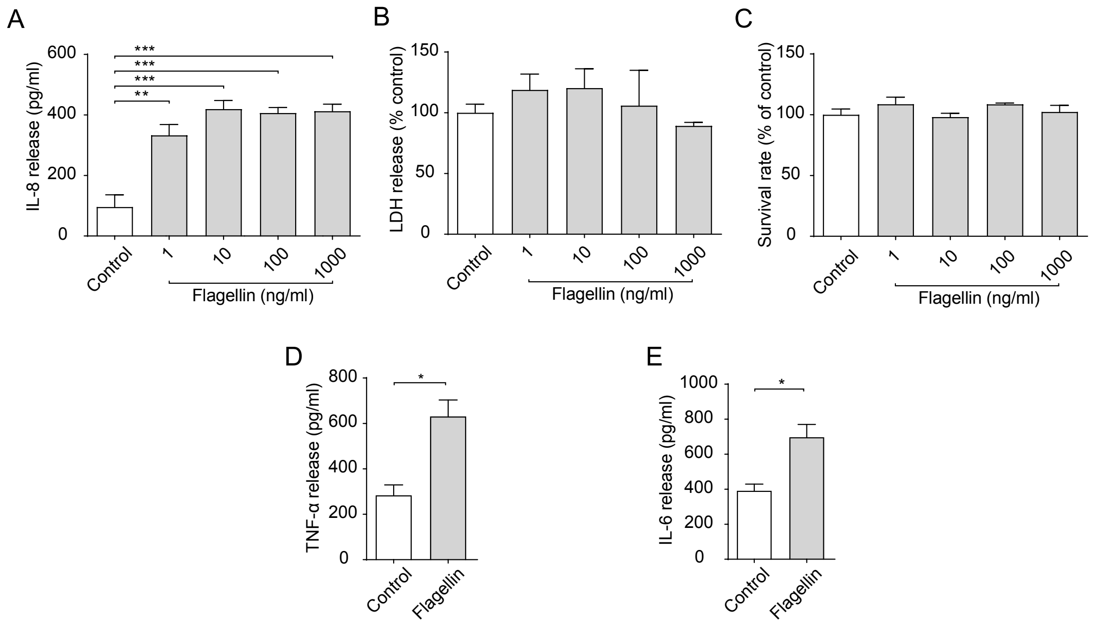

Figure S3. Inflammatory response of PBECs incubated with flagellin. *Passage* 1 (P1) of PBECs were incubated with increasing concentrations of flagellin (1-1000 ng/ml) (**A-C**) for 24 h in the submerged cell culture (SCC) system. (**A)** Interleukin (IL)-8 levels in the supernatants of control and flagellin-treated PBECs were assessed by ELISA. (B) LDH levels were measured in the supernatants and **(C**) survival rates were determined by the percentage of MTT levels in control and flagellin-treated PBECs. (**D-E**) P1 of PBECs were treated with flagellin (10 ng/ml) for 24 h in the SCC system. (**D**) Tumor necrosis factor (TNF)-α and (**E**) IL-6 levels in the supernatants of control and flagellin-treated PBECs were assessed by ELISA. *P<0.05; **P<0.01; ***P<0.001; (**A**–**C**) one-way ANOVA; and (**D-E**) unpaired Student’s t-test. Data are presented as means ± SE (n=3).

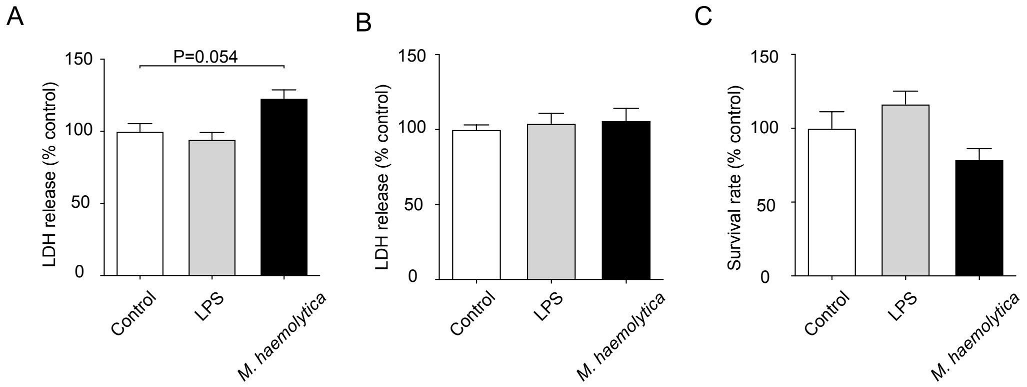

Figure S4. Cytotoxicity effects of *M. haemolytica* and LPS on PBECs. *Passage* 1 (P1) of PBECs were cultured in the liquid-liquid interface (LLI) system and LPS (10 μg/ml) or *M. haemolytica* (1 x10^5 CFU/ml) was added to the apical compartment for 24 h at day 11. LDH release in the supernatants of the apical (**A**) and basolateral (**B**) compartments were measured and (**C**) survival rates were determined by the percentage of MTT levels in control and LPS/*M. haemolytica*-treated PBECs. *P*=0.054 (one-way ANOVA). Data are presented as means ± SE (n=3).

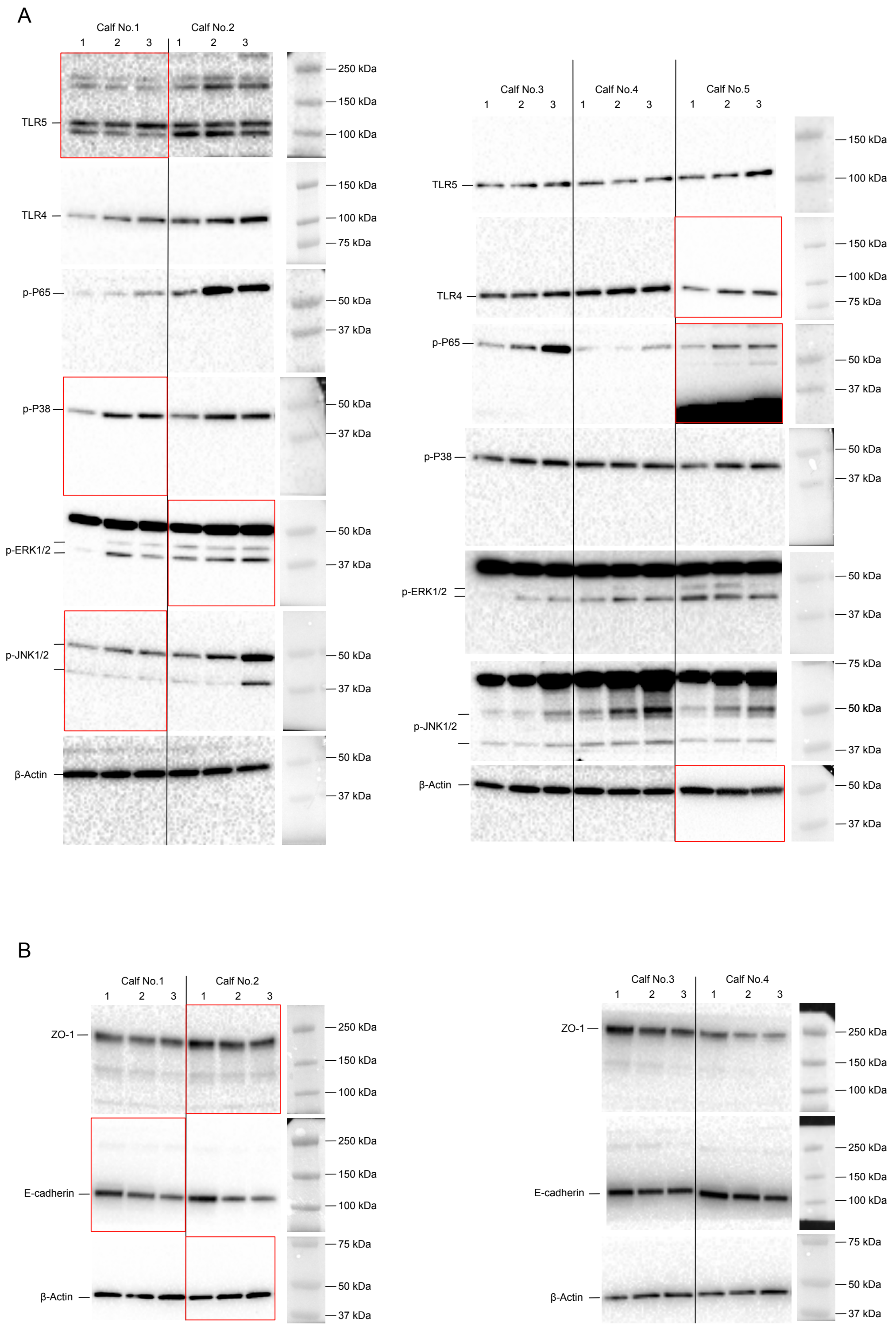

Figure S5. Original blots of figures 4D and 5F.

Lane 1: Control, lane 2: LPS (10 µg/ml) stimulation, lane 3: *M. haemolytica* (1 x10^5 CFU/ml) stimulation. Figure S5A represents the original blots of figure 4D and figure S5B shows the original blots of figure 5F. The original blots with the red boxes represent the cropped images as shown in figure 4D and 5F.
